# Supplementary material for: Transcriptional Profiling of the Small Intestine and the Colon Reveals Modulation of Gut Infection with Citrobacter rodentium According to the Vitamin A Status
Source: Nutrients. 2022 Apr 8;14(8):1563. doi: 10.3390/nu14081563 (PMC9026425; doi:10.3390/nu14081563)
Supplement: Supplementary file 1 [file nutrients-14-01563-s001.zip › nutrients-1651237_supplementary.pdf]

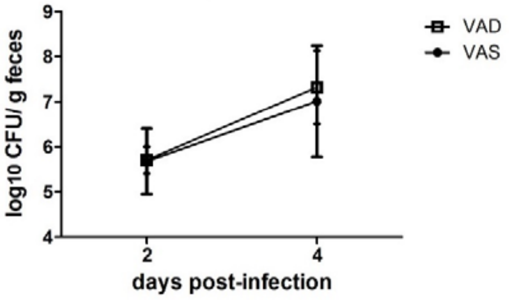

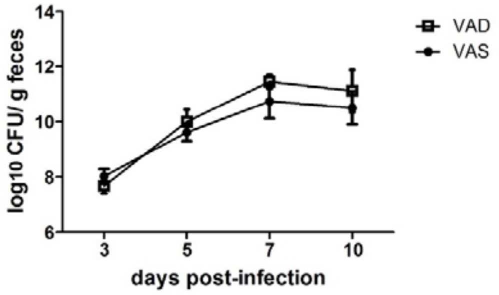

Source of Variation	P value
Interaction	0.6695
VA status	0.3195
Time	< 0.0001

Supplementary Figure S1. *C. rodentium* clearance shows no significant difference between infected VAD and VAS mice during the course of the SI and the colon studies.

C. rodentium bacterial shedding in the feces. (**a**) the SI study stopped on p.i. day 5. Fecal samples were collected twice on p.i. day 2 & day 4 and plated on LB agar plate (with nalidixic acid) for overnight incubation. (**b**) the colon study lasted until p.i. day 10. Feces were collected on p.i. day 3, 5, 7 and 10. Values are the mean ± SEM of n=3-5 mice per group. Two-way ANOVA test with $P<0.05$ as cutoff.
Abbreviation: Colony-forming unit (CFU), vitamin A (VA), vitamin A deficient (VAD), vitamin A sufficient (VAS), small intestine (SI), Luria-Bertani (LB), standard error of the mean (SEM), analysis of variance (ANOVA)

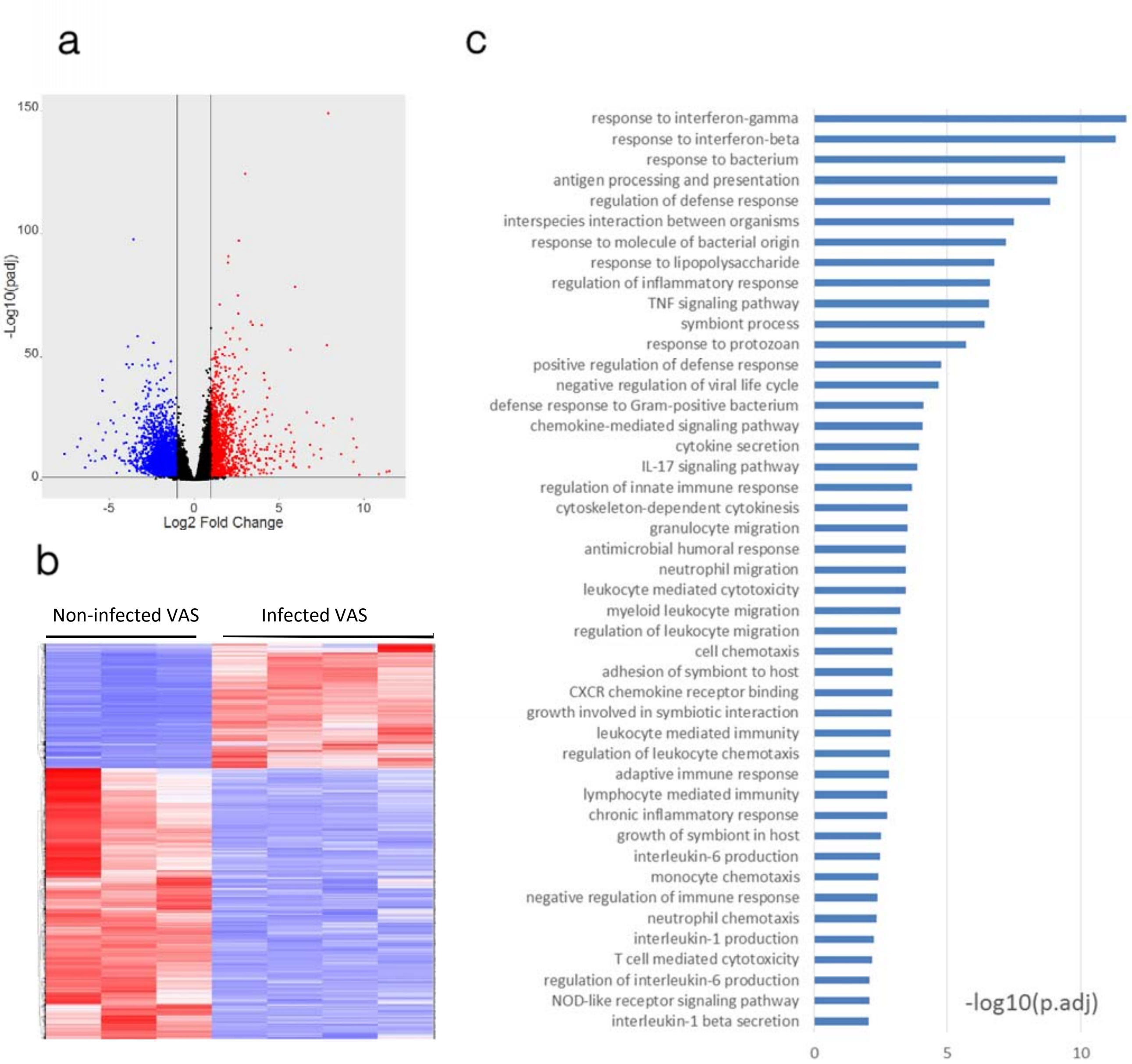

Supplementary Figure S2. Transcriptomic changes corresponding to the VAS-inf in colon study.

(**a**) Volcano plot of DEGs. Red: DEGs upregulated by infection in colon; Blue: DEGs downregulated by infection in colon; Black: non-DEGs. Horizontal line: padj=0.05. Vertical lines: |Fold Change|=2. (**b**) Heatmap of DEGs. Red group: mean expression significantly higher; Blue group: mean expression significantly lower. Non-infected VAS: columns 1, 2 and 3; Infected VAS: columns 4, 5, 6 and 7. (**c**) DEGs upregulated during VAS-inf were enriched in immune-related GO and KEGG categories. Criteria: p.adj<0.05. For a full list of DEGs corresponding to VAS-inf in colon study, see List I in the supplemental Excel file.

Abbreviations: padj: adjusted p value according to DESeq2, differentially expressed gene **(**DEG), vitamin A deficient (VAD), vitamin A sufficient (VAS), Infection effect under VAS status (VAS-Inf), p.adj (BH-adjusted p value from the hypergeometric test conducted by ClusterProfiler), Gene ontology (GO), Kyoto Encyclopedia of Genes and Genomes (KEGG).

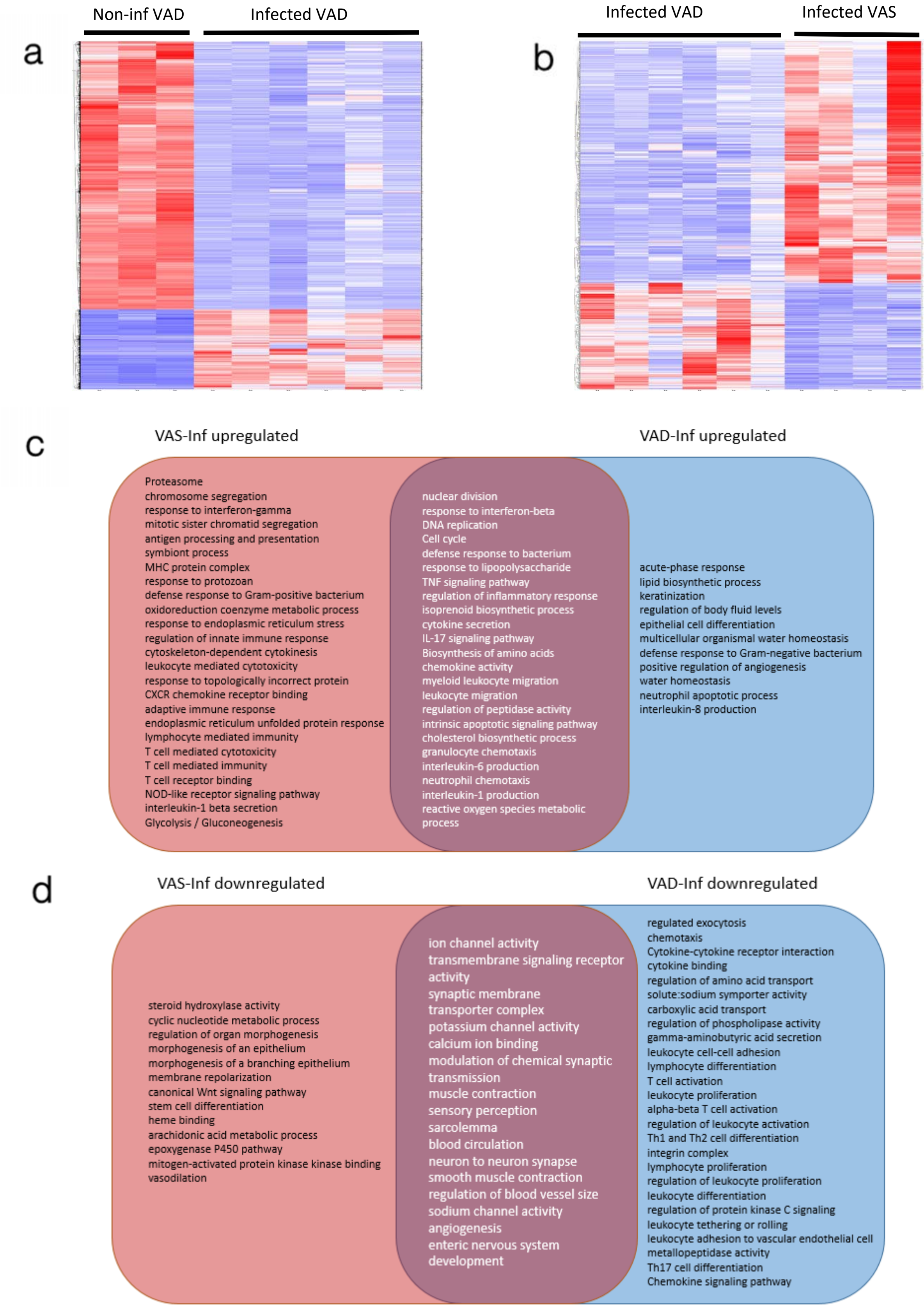
a
Non-inf VAD
Infected VAD
b
Infected VAD
Infected VAS
c
VAS-Inf upregulated
VAD-Inf upregulated
Proteasome
chromosome segregation
response to interferon-gamma
mitotic sister chromatid segregation
antigen processing and presentation
symbiont process
MHC protein complex
response to protozoan
defense response to Gram-positive bacterium
oxidoreduction coenzyme metabolic process
response to endoplasmic reticulum stress
regulation of innate immune response
cytoskeleton-dependent cytokinesis
leukocyte mediated cytotoxicity
response to topologically incorrect protein
CXCR chemokine receptor binding
adaptive immune response
endoplasmic reticulum unfolded protein response
lymphocyte mediated immunity
T cell mediated cytotoxicity
T cell mediated immunity
T cell receptor binding
NOD-like receptor signaling pathway
interleukin-1 beta secretion
Glycolysis / Gluconeogenesis
nuclear division
response to interferon-beta
DNA replication
Cell cycle
defense response to bacterium
response to lipopolysaccharide
TNF signaling pathway
regulation of inflammatory response
isoprenoid biosynthetic process
cytokine secretion
IL-17 signaling pathway
Biosynthesis of amino acids
chemokine activity
myeloid leukocyte migration
leukocyte migration
regulation of peptidase activity
intrinsic apoptotic signaling pathway
cholesterol biosynthetic process
granulocyte chemotaxis
interleukin-6 production
neutrophil chemotaxis
interleukin-1 production
reactive oxygen species metabolic process
acute-phase response
lipid biosynthetic process
keratinization
regulation of body fluid levels
epithelial cell differentiation
multicellular organismal water homeostasis
defense response to Gram-negative bacterium
positive regulation of angiogenesis
water homeostasis
neutrophil apoptotic process
interleukin-8 production
d
VAS-Inf downregulated
VAD-Inf downregulated
steroid hydroxylase activity
cyclic nucleotide metabolic process
regulation of organ morphogenesis
morphogenesis of an epithelium
morphogenesis of a branching epithelium
membrane repolarization
canonical Wnt signaling pathway
stem cell differentiation
heme binding
arachidonic acid metabolic process
epoxygenase P450 pathway
mitogen-activated protein kinase kinase binding
vasodilation
ion channel activity
transmembrane signaling receptor activity
synaptic membrane
transporter complex
potassium channel activity
calcium ion binding
modulation of chemical synaptic transmission
muscle contraction
sensory perception
sarcolemma
blood circulation
neuron to neuron synapse
smooth muscle contraction
regulation of blood vessel size
sodium channel activity
angiogenesis
enteric nervous system development
regulated exocytosis
chemotaxis
Cytokine-cytokine receptor interaction
cytokine binding
regulation of amino acid transport
solute:sodium symporter activity
carboxylic acid transport
regulation of phospholipase activity
gamma-aminobutyric acid secretion
leukocyte cell-cell adhesion
lymphocyte differentiation
T cell activation
leukocyte proliferation
alpha-beta T cell activation
regulation of leukocyte activation
Th1 and Th2 cell differentiation
integrin complex
lymphocyte proliferation
regulation of leukocyte proliferation
leukocyte differentiation
regulation of protein kinase C signaling
leukocyte tethering or rolling
leukocyte adhesion to vascular endothelial cell
metallopeptidase activity
Th17 cell differentiation
Chemokine signaling pathway

Supplementary Figure S3. Transcriptomic changes corresponding to VAD-Inf and the VA effect under infection in colon study.

Heatmaps of DEGs corresponding to the (**a**) VAD-Inf **(**Non-infected VAD: columns 1, 2 and 3; Infected VAD: columns 4 to 9**)** and (**b**) VA effect under infection (Infected VAD: columns 1 to 6; Infected VAS: columns 7 to 10). Red group: mean expression significantly higher; Blue group: mean expression significantly lower. Comparison of representative GO and KEGG terms enriched among upregulated DEGs during infection (**c**) or downregulated DEGs during infection (**d**) between VAS-inf vs VAD-inf. Criteria: p.adj<0.05. For a full list of DEGs corresponding to VAS-inf, VAD-inf, and VA effect under infection, in colon study, see List I, II, and III in the supplemental Excel file.
Abbreviations: differentially expressed gene **(**DEG), vitamin A deficient (VAD), vitamin A sufficient (VAS), Gene ontology (GO), Kyoto Encyclopedia of Genes and Genomes (KEGG), Infection effect under VAS status (VAS-Inf), Infection effect under VAD status (VAD-Inf). p.adj: BH-adjusted p value from the hypergeometric test done by ClusterProfiler.

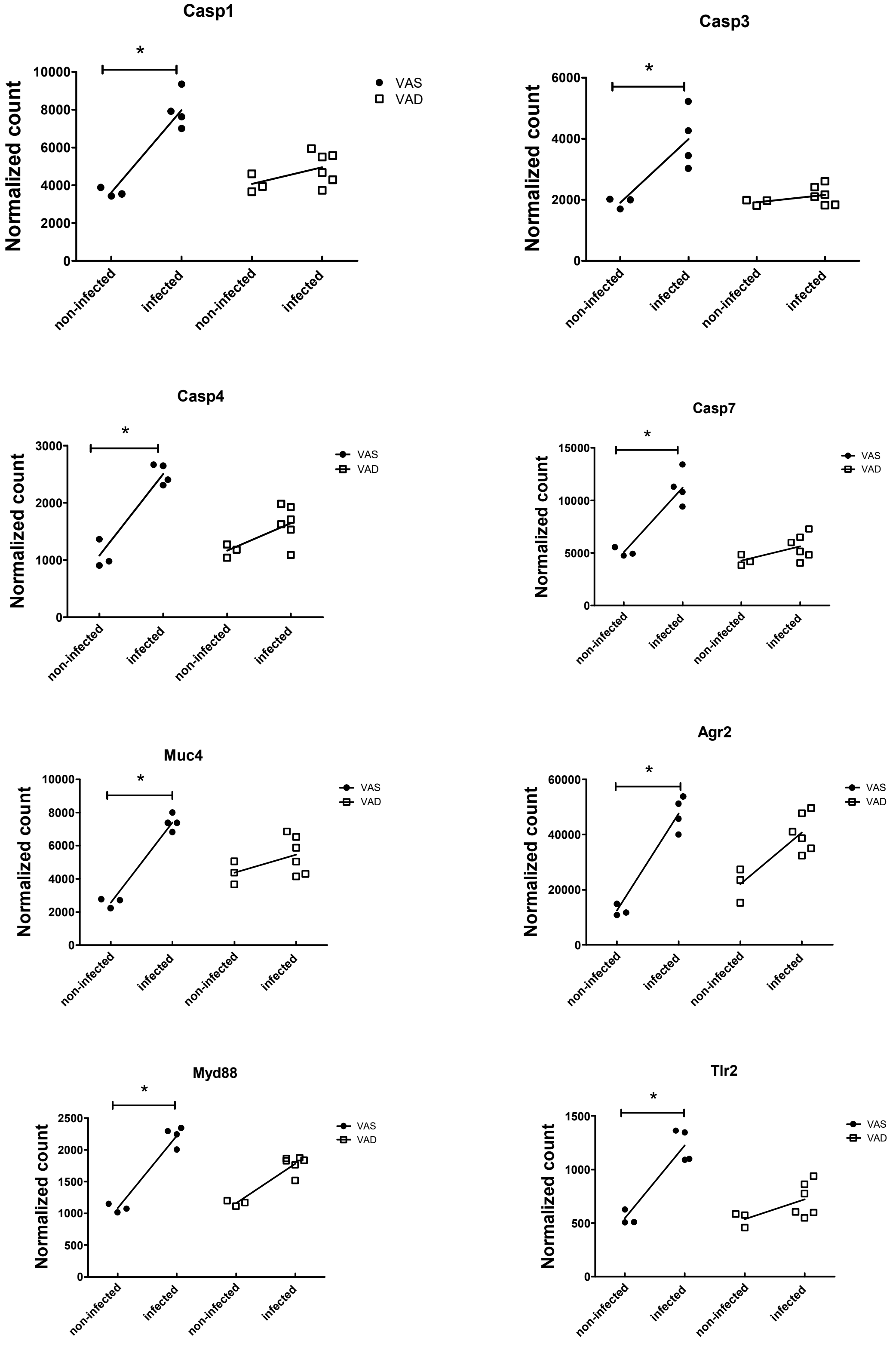
Casp1
Casp3
Casp4
Casp7
Muc4
Agr2
Myd88
Tlr2
Normalized count
*
VAS
VAD
non-infected
infected

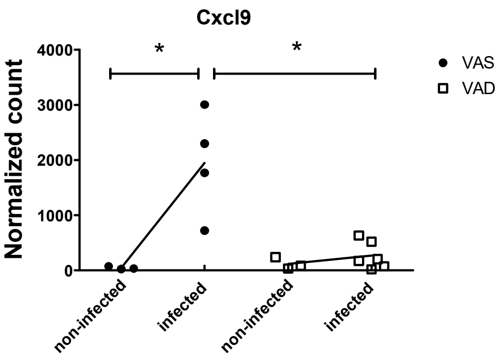

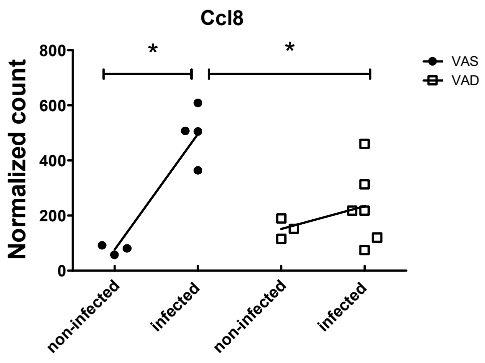

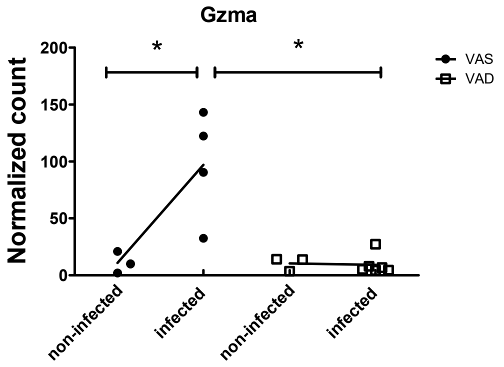

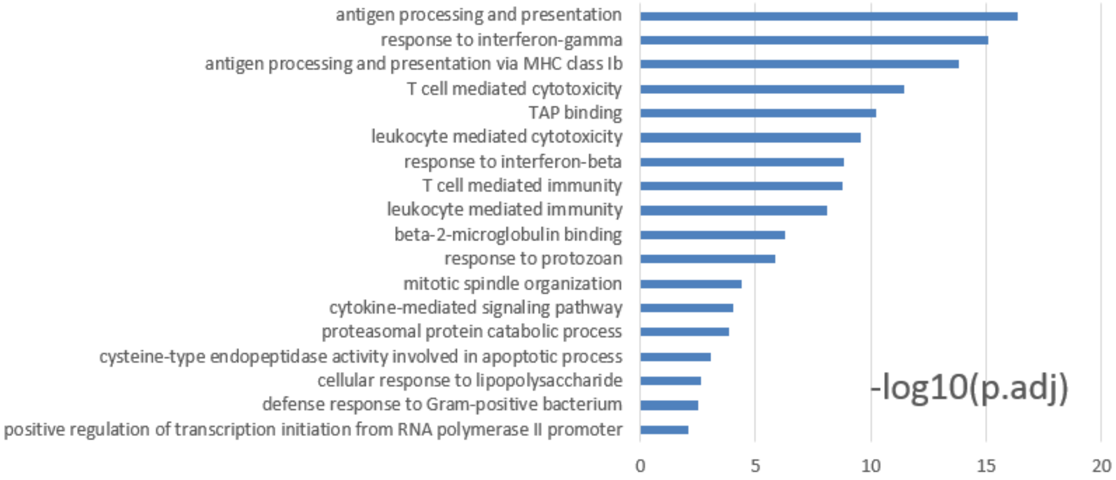

Supplementary Figure S4. Transcriptomic changes in Scenario 1.

(**a-k**) Representative DEGs corresponding to Scenario 1 in the colon study. *in the differential expression analysis, adjusted p value <0.05 and |FoldChange|>2.

(**l**) GO and KEGG enrichment terms of DEGs corresponding to Scenario 1 in the colon study. Criteria: p.adj<0.05. For a full list of genes corresponding to Scenario 1 of the Interaction effect, see Scenario 1 in the supplemental Excel file.

Abbreviations: differentially expressed gene (DEG), vitamin A deficient (VAD), vitamin A sufficient (VAS), p.adj (BH-adjusted p value from the hypergeometric test conducted by ClusterProfiler), Gene ontology (GO), Kyoto Encyclopedia of Genes and Genomes (KEGG).

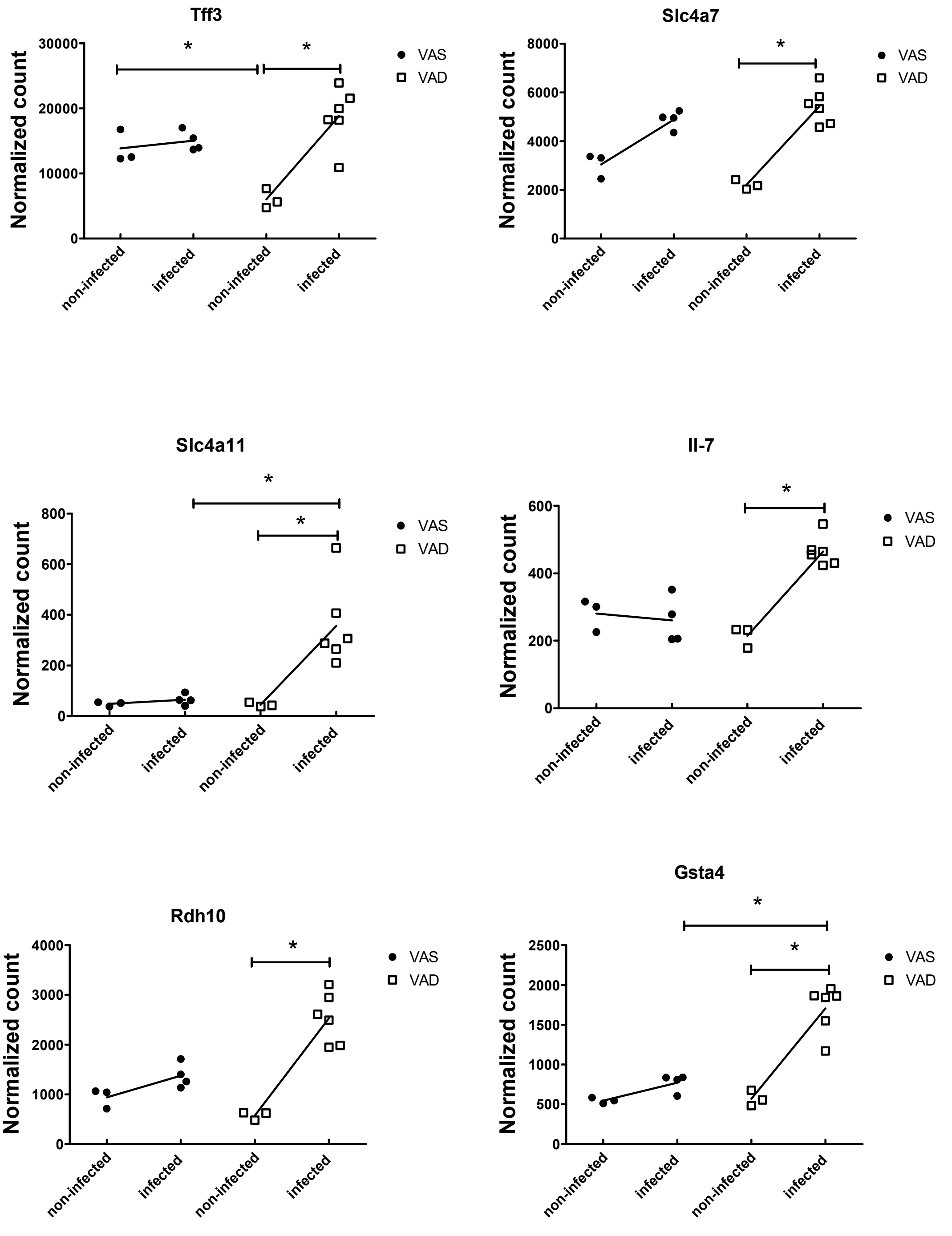
Tff3
Slc4a7
Slc4a11
Il-7
Rdh10
Gsta4
Normalized count
non-infected
infected
VAS
VAD
*
30000
20000
10000
8000
6000
4000
2000
800
600
400
200
2500
1500
1000
500
0

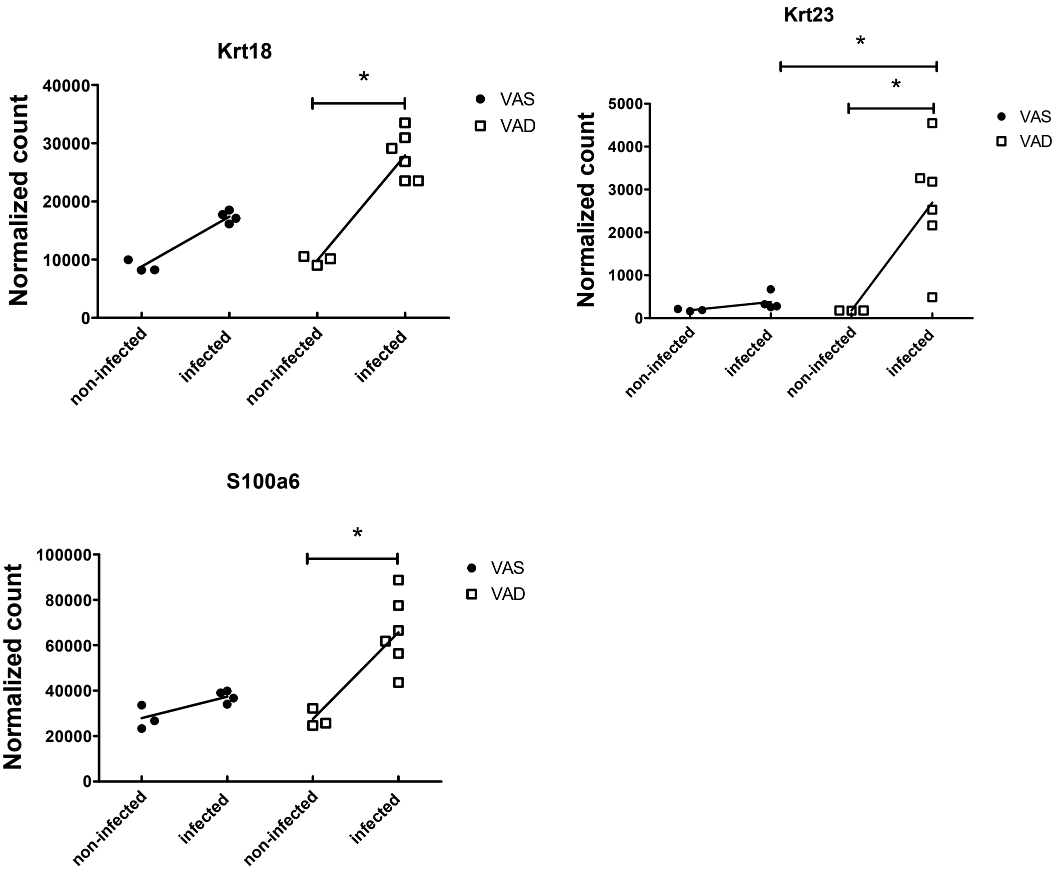

Supplementary Figure S5. Representative DEGs corresponding to Scenario 3 in the colon study.

*in the differential expression analysis, adjusted p value <0.05 and |FoldChange|>2. For a full list of genes corresponding to Scenario 3 of the Interaction effect, see Scenario 3 in the supplemental Excel file. **Abbreviations:** differentially expressed gene **(**DEG), vitamin A deficient (VAD), vitamin A sufficient (VAS).

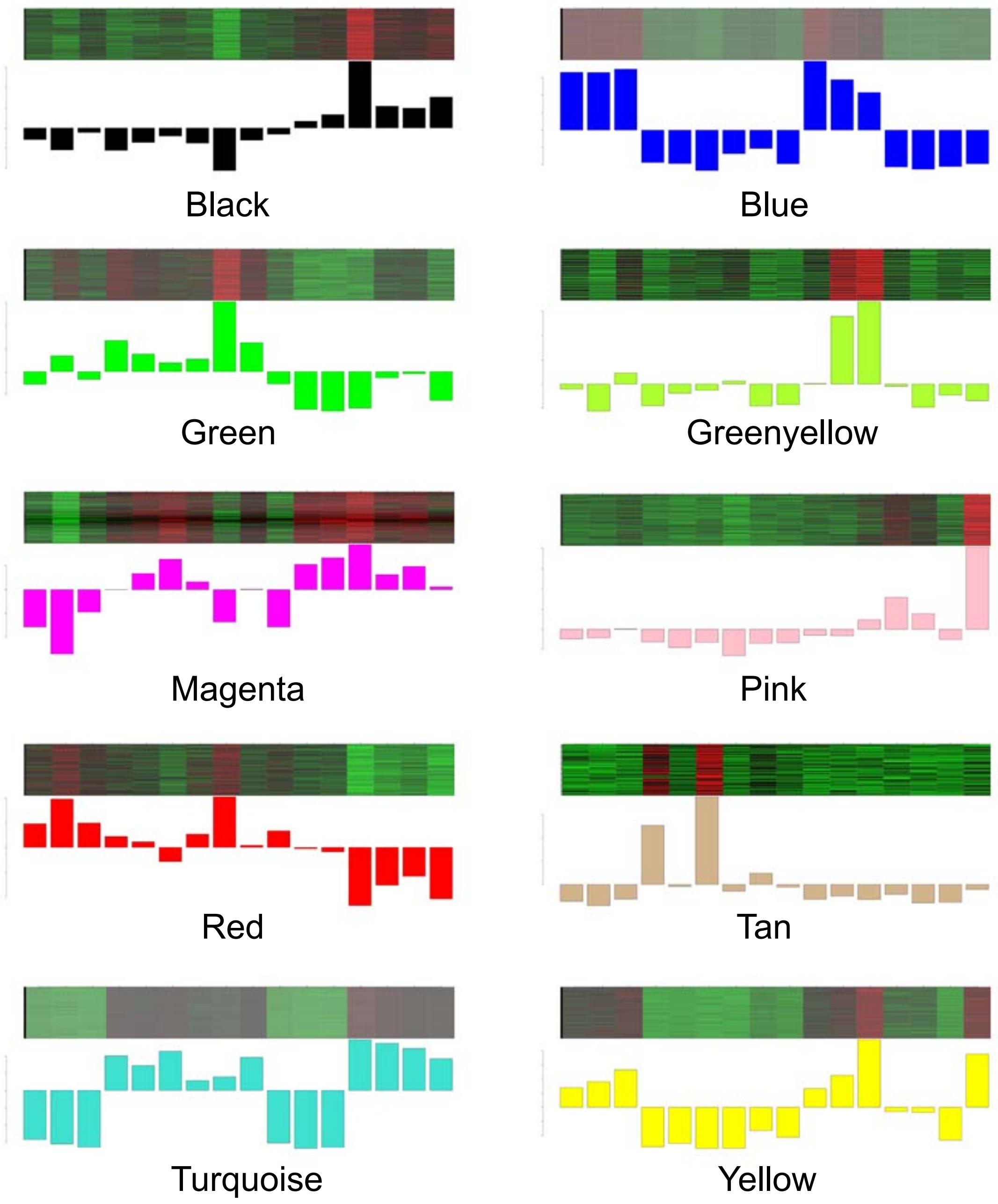

Supplementary Figure S6. Module eigengenes in colon WGCNA network. Eigengenes values (Y axis) of modules across samples (X axis) are visualized. Purple and Brown modules are shown in Figure 6. For full lists of genes of each modules, see supplemental Excel file. In each heatmaps, Red: Higher expression; Green: Lower expression. non-infected VAD: columns 1, 2, and 3. Infected VAD: columns 4, 5, 6, 7, 8, and 9. non-infected VAS: columns 10, 11, and 12. Infected VAS: 13, 14, 15, and 16.

Supplementary Table S1. Immune- and epithelial-related DEGs upregulated and downregulated in VAS-Inf in colon study

Infected VAS group (n=4) vs non-infected VAS group (n=3). The order is ranked alphabetically by the Official Gene Symbol. DEGs are organized into immune cell-related and epithelial cell-related, the two major cell categories in the intestine. For a full list of DEGs corresponding to VAS-inf in colon study, see List I in the supplemental Excel file.

Abbreviations:
Differentially expressed gene **(**DEG)
Infection effect under VAS status (VAS-Inf)
BaseMean: Mean expression level (normalized by DESeq2) across all 16 libraries
Fold Change: Normalized mean expression level of Infected VAS group / Normalized mean expression level of non-infected VAS group
padj: adjusted p value calculated by DESeq2

DEGs upregulated in VAS-Inf in colon study

Immune cell-related

	baseMean	FoldChange	padj	ENTREZID	Fullname
Casp1	5288.81	2.21	6.10E-13	12362	caspase 1
Casp3	2524.49	2.09	1.67E-09	12367	caspase 3
Casp4	1664.04	2.32	1.74E-10	12363	caspase 4 apoptosis-related cysteine peptidase
Casp7	6669.61	2.21	3.34E-10	12369	caspase 7
Casp8	4625.84	2.56	2.12E-26	12370	caspase 8
Ccl2	66.03	9.22	8.88E-08	20296	chemokine (C-C motif) ligand 2
Ccl3	30.48	6.07	3.56E-02	20302	chemokine (C-C motif) ligand 3
Ccl7	147.46	15.32	2.88E-13	20306	chemokine (C-C motif) ligand 7
Ccl8	254.30	6.46	1.82E-09	20307	chemokine (C-C motif) ligand 8
Ccl9	585.17	2.38	9.26E-19	20308	chemokine (C-C motif) ligand 9
Cxcl1	173.53	22.74	1.10E-18	14825	chemokine (C-X-C motif) ligand 1
Cxcl10	324.91	8.10	1.29E-04	15945	chemokine (C-X-C motif) ligand 10
Cxcl11	24.02	14.60	8.57E-09	56066	chemokine (C-X-C motif) ligand 11
Cxcl2	168.47	57.78	2.40E-07	20310	chemokine (C-X-C motif) ligand 2
Cxcl3	52.33	665.37	2.34E-08	330122	chemokine (C-X-C motif) ligand 3
Cxcl5	1697.03	197.90	8.09E-23	20311	chemokine (C-X-C motif) ligand 5
Cxcl9	618.94	43.85	1.45E-08	17329	chemokine (C-X-C motif) ligand 9
Cxcr2	27.50	7.82	2.03E-03	12765	chemokine (C-X-C motif) receptor 2
Fcgr1	86.01	2.28	3.56E-05	14129	Fc receptor IgG high affinity I
Gprc5a	9824.63	2.99	7.65E-15	232431	G protein-coupled receptor family C group 5 member A
Il11	40.79	4.59	3.82E-04	16156	interleukin 11

Il18bp	725.08	11.29	3.86E-25	16068	interleukin 18 binding protein
Il18rap	88.72	2.38	5.03E-03	16174	interleukin 18 receptor accessory protein
Il1f9	23.31	64.18	4.95E-06	215257	interleukin 1 family member 9
Il1r2	52.24	4.24	1.42E-03	16178	interleukin 1 receptor type II
Il1rl1	163.50	5.34	9.57E-06	17082	interleukin 1 receptor-like 1
Il4ra	2680.26	2.59	8.64E-14	16190	interleukin 4 receptor alpha
Lcn2	318.99	35.26	2.39E-15	16819	lipocalin 2
Mmp19	570.07	3.34	9.38E-15	58223	matrix metallopeptidase 19
Mmp28	505.07	4.33	2.62E-17	118453	matrix metallopeptidase 28 (epilysin)
Mmp3	181.18	3.55	8.49E-05	17392	matrix metallopeptidase 3
Mmp7	198.28	42.39	1.98E-21	17393	matrix metallopeptidase 7
Myd88	1642.45	2.06	1.49E-30	17874	myeloid differentiation primary response gene 88
Nipal1	1415.55	2.23	5.31E-11	70701	NIPA-like domain containing 1
Nlrc5	1218.75	6.76	1.78E-08	434341	NLR family CARD domain containing 5
Nod2	211.34	3.36	1.48E-11	257632	nucleotide-binding oligomerization domain containing 2
Pdpn	775.98	2.99	2.54E-08	14726	podoplanin
Pycard	5977.48	2.10	2.14E-14	66824	PYD and CARD domain containing
Reg3b	4183.62	131.67	4.04E-11	18489	regenerating islet-derived 3 beta
Reg3g	2392.01	59.81	2.00E-12	19695	regenerating islet-derived 3 gamma
S100a10	14623.21	2.74	4.31E-16	20194	S100 calcium binding protein A10 (calpactin)
S100a11	10864.21	3.79	3.98E-31	20195	S100 calcium binding protein A11
S100a14	12448.46	4.88	1.68E-62	66166	S100 calcium binding protein A14
S100a16	14187.12	3.92	6.31E-89	67860	S100 calcium binding protein A16
S100a8	1492.11	631.53	2.08E-25	20201	S100 calcium binding protein A8 (calgranulin A)
S100a9	1558.94	673.48	1.85E-17	20202	S100 calcium binding protein A9 (calgranulin B)
Saa2	24.97	3.60	2.80E-03	20209	serum amyloid A 2
Saa3	673.21	11.65	1.07E-06	20210	serum amyloid A 3
Saa4	34.08	13.93	5.23E-05	20211	serum amyloid A 4
Tlr2	780.49	2.24	2.04E-09	24088	toll-like receptor 2
Tnf	241.16	4.98	9.04E-04	21926	tumor necrosis factor
Tnfaip2	472.93	2.18	9.75E-04	21928	tumor necrosis factor alpha-induced protein 2
Tnfaip3	8635.85	4.13	6.13E-16	21929	tumor necrosis factor alpha-induced protein 3
Tnfaip8	2231.86	2.22	2.30E-23	106869	tumor necrosis factor alpha-induced protein 8
Tnfrsf12a	617.11	2.08	9.83E-03	27279	tumor necrosis factor receptor superfamily member 12a

Tnfrsf1a	4355.18	2.65	7.14E-39	21937	tumor necrosis factor receptor superfamily member 1a
Tnfrsf1b	3441.58	5.51	2.61E-34	21938	tumor necrosis factor receptor superfamily member 1b
Tnfrsf23	250.77	5.29	3.00E-10	79201	tumor necrosis factor receptor superfamily member 23
Tnfrsf8	253.70	13.66	2.54E-28	21941	tumor necrosis factor receptor superfamily member 8
Tnfsf10	1163.77	4.06	9.08E-09	22035	tumor necrosis factor (ligand) superfamily member 10
Tnfsf9	87.63	2.79	4.89E-05	21950	tumor necrosis factor (ligand) superfamily member 9

DEGs upregulated in VAS-Inf in colon study

Epithelial cell-orginated

	baseMean	FoldChange	padj	ENTREZID	Fullname
Aacs	5184.43	2.18	2.88E-08	78894	acetoacetyl-CoA synthetase
Actb	109598.47	2.33	2.46E-30	11461	actin beta
Agr2	33626.68	3.82	5.46E-22	23795	anterior gradient 2
Ak4	1004.18	4.72	4.91E-09	11639	adenylate kinase 4
Areg	1083.09	4.96	2.88E-16	11839	amphiregulin
Barx2	188.38	2.57	8.34E-13	12023	BarH-like homeobox 2
Ccnd1	4755.04	3.16	2.61E-12	12443	cyclin D1
Cd177	17774.21	4.94	1.21E-29	68891	CD177 antigen
Cd274	464.99	2.25	9.97E-04	60533	CD274 antigen
Cd38	12742.75	3.32	1.56E-17	12494	CD38 antigen
Cebpd	1316.13	2.40	9.24E-07	12609	CCAAT/enhancer binding protein (C/EBP) delta
Ckap4	3904.11	6.18	1.14E-45	216197	cytoskeleton-associated protein 4
Clca3a1	692.05	2.09	3.31E-06	12722	chloride channel accessory 3A1
Clca3a2	1456.17	3.26	1.96E-07	80797	chloride channel accessory 3A2
Clca3b	37907.11	5.23	7.44E-20	229927	chloride channel accessory 3B
Clca4a	4326.95	2.02	3.74E-02	99663	chloride channel accessory 4A
Clca4b	35258.66	100.00	2.54E-28	99709	chloride channel accessory 4B
Cldn7	27621.70	2.20	9.32E-25	53624	claudin 7
Cldn8	4811.96	2.13	7.43E-04	54420	claudin 8
Duox2	11343.50	3.22	1.86E-06	214593	dual oxidase 2
Duoxa2	4801.28	6.12	4.41E-15	66811	dual oxidase maturation factor 2
E2f2	2370.11	8.17	4.60E-44	242705	E2F transcription factor 2
Fdps	6681.29	4.37	3.98E-58	110196	farnesyl diphosphate synthetase
Fut2	9180.17	6.02	2.14E-68	14344	fucosyltransferase 2

Gbp2	4786.18	9.23	6.75E-13	14469	guanylate binding protein 2
Glt28d2	345.83	2.18	1.22E-06	320302	glycosyltransferase 28 domain containing 2
Gml	60.49	175.86	1.62E-13	625599	glycosylphosphatidylinositol anchored molecule like
Gzma	31.73	8.94	5.12E-05	14938	granzyme A
Ifi35	1134.17	2.03	2.37E-15	70110	interferon-induced protein 35
Igtp	2877.98	19.70	3.22E-14	16145	interferon gamma induced GTPase
Iqgap2	13155.93	2.36	6.40E-11	544963	IQ motif containing GTPase activating protein 2
Lrg1	11239.83	238.75	9.28E-150	76905	leucine-rich alpha-2-glycoprotein 1
Mnda	49.11	3.26	2.64E-06	NA	NA
Muc1	1208.43	4.63	1.67E-13	17829	mucin 1 transmembrane
Muc2	84214.41	2.45	1.69E-04	17831	mucin 2
Muc4	5190.55	2.88	1.39E-17	140474	mucin 4
Nos2	5879.07	59.26	2.49E-16	18126	nitric oxide synthase 2 inducible
Ntn5	42.58	3.06	1.18E-05	243967	netrin 5
Pla2g5	1764.10	5.23	1.09E-50	18784	phospholipase A2 group V
Plet1	14962.81	226.92	1.69E-55	76509	placenta expressed transcript 1
Prss27	113.34	18.41	1.92E-29	213171	protease serine 27
Rab27a	1406.10	2.57	3.07E-16	11891	RAB27A member RAS oncogene family
Retnlb	4272.92	10.26	1.18E-07	57263	resistin like beta
Smpdl3b	632.52	5.14	1.27E-25	100340	sphingomyelin phosphodiesterase acid-like 3B
Socs1	829.81	19.37	1.24E-26	12703	suppressor of cytokine signaling 1
Stat1	5491.46	6.36	7.76E-17	20846	signal transducer and activator of transcription 1
Stat2	2690.94	2.90	2.71E-18	20847	signal transducer and activator of transcription 2
Syt8	1081.81	2.73	2.74E-04	55925	synaptotagmin VIII
Vil1	25271.06	2.18	2.14E-15	22349	villin 1
Xbp1	9698.52	2.33	2.12E-32	22433	X-box binding protein 1
Zbp1	2292.43	11.12	1.79E-22	58203	Z-DNA binding protein 1

DEGs downregulated in VAS-Inf in colon study

Immune cell-related

	baseMean	FoldChange	padj	ENTREZID	Fullname
Ahrr	22.12	-2.29	3.23E-02	11624	aryl-hydrocarbon receptor repressor
Mmp11	122.96	-6.40	4.41E-40	17385	matrix metallopeptidase 11
Mmp15	4401.80	-2.00	2.07E-06	17388	matrix metallopeptidase 15
Mmp16	22.15	-5.00	8.78E-07	17389	matrix metallopeptidase 16
Mmp17	292.05	-2.73	1.09E-04	23948	matrix metallopeptidase 17
Mmp23	169.01	-2.62	1.52E-09	26561	matrix metallopeptidase 23
Mmp24	19.00	-2.93	3.65E-03	17391	matrix metallopeptidase 24
Timp2	2714.83	-3.07	5.13E-12	21858	tissue inhibitor of metalloproteinase 2
Timp4	126.76	-2.55	7.88E-07	110595	tissue inhibitor of metalloproteinase 4

DEGs downregulated in VAS-Inf in colon study

Epithelial cell-orginated

	baseMean	FoldChange	padj	ENTREZID	Fullname
Angpt1	54.58	-2.92	1.63E-09	11600	angiopoietin 1
Cldn1	47.37	-2.27	2.08E-02	12737	claudin 1
Cldn5	128.73	-5.38	2.85E-08	12741	claudin 5
Id4	390.89	-3.75	1.61E-13	15904	inhibitor of DNA binding 4
Lgr5	457.90	-2.59	7.46E-04	14160	leucine rich repeat containing G protein coupled receptor 5
Nkx2-2	51.15	-2.30	8.93E-07	18088	NK2 homeobox 2
Rspo2	20.23	-2.88	6.04E-03	239405	R-spondin 2
Rspo3	144.49	-3.54	8.48E-05	72780	R-spondin 3

Supplementary Table S2. Colon modules and their sizes, functional annotations, hub genes, and overlap with DEGs in the VAS-Inf comparison

Abbreviations:

Differentially expressed gene **(**DEG)

MS: Module Size. Number of gene in certain modules.

DEG count: number of DEGs (corresponding to the Infection effect) that also belong to certain module

DEG ratio: DEG count/MS

Selective DEGs: DEGs that are also top 5% hub genes

Module color	MS	Representative functional annotations	Top 5% Hub Genes (ranked by connectivity)	DEG count	DEG ratio %
black	246	MHC protein complex	Tmem106a Cxcr3 Neat1 Gpr55 Rdh16 Ciita Dram2 Mgat1 Slc37a1 Ccdc116 H2-T23 Ppp6r1 Agpat1	5	2.03%
blue	5674	chemical synaptic transmission, axon development, regulation of transmembrane transport, extracellular matrix, cation channel complex	Prrc2b Tbccd1 Hoxd12 Fhit Zmiz1 Tusc5 Syde2 Syn3 Gng3 1700001L05Rik Unc80 Ulk1 Chrna5 Tshz1 Zfp516 Raver2 Slc2a4rg-ps Trim44 Rprml Mrc2 Dnah5 Glis2 Ccdc157 Kazald1 Wdtc1 Dpysl3 Foxp1 Casp9 Plekha4 Ccdc15 Tmem237 Zscan18 Sesn3 Acadvl Ephx4 Camk2g 1700109H08Rik Cox7a2l Fendrr Fam193b Flt3l Rab11fip5 9330182L06Rik Fzd2 Kif3c Nkx2-2 C920006O11Rik Ddrgk1 Fabp2 Ccdc57 Adam19 Kxd1 Fn3k Gsdmc2 Gm11240 Lix1 Pcnx Fbn1 Vwa1 Tob2 Pcdhb16 Rab4a Fam189a2 Pou2f3 Igf1 Rab30 Nudt16 Tgfbi 1300002E11Rik Trpm5 Mapk8ip3 Ago3 Nr2c2 Car15 Col9a2 Anks1 Zcchc18 Slitrk1 Ism1 Usp20 Plekho2 Gm4262 Plxnb3 Lca5 Col25a1 Slu7 Nhlrc1 2610044O15Rik8 Eci3 Mmp2 Zfp846 Capn3 Cav3 Zfp346 Ppic Mir678 Tmem163 Arhgap20 Sema3d P4htm 9230114K14Rik Asphd2 Add2 Vezf1 Zfp592 Pds5b Adh1 D430019H16Rik Acad10 Klhl36 Stra6l Gpank1 Zfp595 Crb2 Casq2 Efhd1 Coro2b Ulk2 Fgfrl1 Pcgf1 Musk Htr3a Cacna1c Tmem132a Zfp94 Map3k3 4930511M06Rik Ypel1 Slc45a1 Ctnnd2 Tomm6os Emp3 1110002L01Rik Acss1 Rgs7 Rhebl1 1010001N08Rik Susd1 Rab36 Slc26a11 4930402H24Rik Hip1 Npy4r Resp18 Gm12359 Trim62 Rsl1 Hrg Col11a2 Sh3gl2 Wnt6 Pcdh20 Bcl9 Hint1 Atg10 Nr1h5 Recql5 Kcnv1 Tet1 1700123I01Rik Plekha5 Lrp12 Gm6277 Hoxa5 Adgrb3 Scamp5 Car3 Aamdc Slc7a2 Zfp748 Syt7 Fbxl16 Tbkbp1 Chd5 Sh3rf3 Zfp316 Cntln 2510009E07Rik Slc24a3 Meis1 Tceanc2 Jakmip3 Lrp3 Edn1 Itm2b Zfp629 Khk Flywch1 Cdyl2 Cyp2f2 Pank4 Dcdc2b Arhgef37 Numbl Zfyve28 Slc10a4 Eppk1 Fam184a Ces2c Mettl7b Zmat1 Dcp1b Trpm3 Cmklr1 Sdc4 Spata2l Eng Cilp Tubal3 Gm10336 Atad3aos Cep131 Arhgap10 Zfp108 Tril Ucp3 6430573F11Rik Prune2 Fam163a Id4 C1qtnf4 Il17d Eif2ak4 Lrsam1 Fcgrt Sez6l Oaz3 Stk17b Ccbe1 Xpc Pard3b Cplx1 Zkscan8 Ggn Ptrf Sepp1 Abca8a 1700037H04Rik Socs7 Pde4a Zeb2os Tmem8b Fez1 Gabra3 Fbln2 Sulf1 Mapt Zfp141 Fat2 Map7d2 Sfrp1 Tprgl Angptl1 Ilvbl Tspyl5 Cap2 Inafm1 Hist1h1e Ddit4l Zfp157 Sepn1 Nsg2 Plcd4 Cml1 Maf Ndn Hcst Anxa6 Prmt2 Lrrc27 Zfp956 Rassf10 Gm20257 Ptgir Ugt2b35 Araf Arap3 Plekhb1 Bend4 2310001H17Rik Zfp638 Zfp329 Ryr3 Hexdc	2967	52.29%
brown	577	-	Tmem43 Rnf39 Elmo3 Nfatc2 Pygo2 Fbxo11 Saa3 Mmp10 Fbxo11 Tlcd2 Timp1 Brap Mink1 Liph Tmem41a Wdr26 Med15 Timp1 Zbtb48 Dok7 Trim29 Ano6 Zfc3h1 Adnp Polk Zfp322a Stx19 Krt7	89	15.42%
green	466	cytoplasmic translation, ribosome	Mir7079 Pitrm1 Reps1 Slc38a10 Dtnbp1 Ptpro Rad51b Mfsd6l Rcl1 Ppp2cb Pdcd2 Wdr53 Polr3h Rps10	9	1.93%

		biogenesis, ncRNA processing, rRNA metabolic process, mRNA binding, nuclease activity	Hmga1-rs1 Snapc4 Snhg8 G6pc3 Ewsr1 Lsm11 Adh5 Ewsr1 Ccdc14 Fhl3		
greenyellow	174	transcription cofactor activity, transcription factor activity, protein binding, proximal promoter sequence-specific DNA binding	Dbp Ncoa4 Cipc Ccbl1 Trp53inp1 Rest Zswim6 Fam126b Fam76a	20	11.49%
grey	897	-	-	8	0.89%
magenta	191	-	Stard4 Phf20l1 Eif2ak1 Mob1b Amfr Brd1 Arl6ip6 Hpse Dyrk1a Zfp407	0	0%
pink	241	leukocyte mediated immunity, adaptive immune response, lymphocyte mediated immunity, leukocyte migration, response to bacterium, T cell activation, neutrophil activation, phagocytosis, B cell mediated immunity	Mx2 Havcr2 Cxcl10 Plek Fam177a Tigit Ctrl Crlf3 Ocstamp Mzb1 2310045n01rik-mef2b Egr2	58	24.07%
purple	184	anion transmembrane transporter activity	Xrn1 Rasef Ptgr2 Dgkd Tmtc2 Clic5 Akap10 Slc7a9 Ush1c Lrp6	0	0%
red	258	-	Gjb3 Efna4 Cln6 Ttc26 Ngrn Smim5 S100g Fmo1 2010320M18Rik Cspp1 Rgcc Parp1 Smoc2	25	9.69%
tan	87	positive regulation of secretion by cell, cytokine secretion	Gm14440 Il1b Galnt6 Xkr4 Arg2	12	13.79%
turquoise	5799	mRNA processing, ncRNA metabolic process, chromosome segregation, ribosome biogenesis, RNA splicing, mitochondrion organization, establishment of protein localization to organelle, Golgi vesicle transport, tRNA metabolic process, ubiquitin-dependent protein catabolic process	Xpnpep1 Psmd2 Pmpca Psmd6 Pde12 Ywhae Tpm3 Metap2 Ppp1ca Phf5a Morf4l2 Lrrc47 Mphosph6 Nars Txnl1 Ddah1 Itpa Tor2a Lsm12 Ccdc43 Tars Pmm2 Ykt6 Mta2 Rad23b Etf1 Gnb2 Plaa Golt1b Ciapin1 Bzw1 Gars Ddx39 Eif5a Pgd Ap3m1 Med8 Rpl7l1 Ccdc124 Prelid1 Dera Cct6a Ybx1 Psme3 P4hb Hsd17b12 Mrpl4 Dnajc10 Tmem79 Chmp6 Nudcd2 Psmd13 Mrpl12 Taf2 H2afz Mrpl18 Psma3 Gorasp2 Prdx1 Tomm22 Cul2 Rab8a Cfl1 E2f4 Ube2n Shkbp1 Hmbs Tmed9 Ap2m1 Lamtor1 Kctd5 Vcp Ap1s1 Ube2l3 Smu1 Hn1l Zdhhc3 Gtf2f2 Psmc3 Actr1a Ttc13 Gspt1 Cdv3 Keap1 Fdps Rars Tmem14c Lsm6 Gmds Tfdp1 Farsa Psmd14 Dnaaf5 Cltb Slc35a4 Scamp4 Ralb Aars Ppih Ppp2r1b Rps6ka1 Gnb1 Crcp Psmd11 Tomm40 Srp72 Pmvk Psmc1 Psmc2 Lsm4 Slc30a7 Hnrnpm Mrpl36 Hspa14 Larp1b Psmd1 Afg3l1 Atpaf2 Dnajc11 Tmed2 Ccdc109b Psmd7 Pdap1 Dpp3 Anxa4 Nmt1 Hars Pkp2 Mrpl37 Sub1 Clic1 Ergic2 Arhgdia Cstf3 Srp9 Tuba1c Mydgf Ostc Dazap1 Syngr2 Nckap1 Ywhah Psmc6 Tuba4a Mrpl10 Exosc3 Med10 Eif4g1 Glo1 Eif4e Rqcd1 Ccar2 Mrpl47 Cdkn2aipnl Aggf1 Naa15 Mrps7 Dcaf13 Cdc34 Mrpl21 Bpgm Ywhaz Dpp9 Srsf9 Ccdc115 Klhdc4 Snrpb2 Psmc4 Mvp Pdcd6 Rbm14 Traf7 Nme1 Prpf38a Fam207a Ube2k Metap1 Eif2b5 Slc35c1 Rab1 Pigk Tmem126a Usp5 Ssr1 Ccdc25 Mrpl46 Hdac1 Ppp2r1a Nr2c2ap Lsm3 Ttc39a Ncbp1 Prelid2 Mrps25 Ythdf2 E2f2 Ptk2 Smarcb1 Gsr Sf3a1 Dync1li1 Bud31 Eif1ad Mrpl20 Pdia3 B3gnt3 Bbip1 Cog2 Akt1 Gdi2 Akr1a1 Pgs1 Cars Pcbp1 Rab35 Psmd8 Ppp4c Mrpl11 Mrps14 Nol7 Dolpp1 Ripk3 Mmadhc Acsl5 Strap Nadk Adrm1 Hspe1 Eno1b Znhit1 Casp8 Eno1 Psma2 Jagn1 Psmd3 Uchl3 Psmd12 Med19 Lrrc59 Asna1 Nudt5 Gnai3 Cpne2 Copb2 Prkrip1 Cops5 Atxn7l3 Ltbr Hspa4	1255	21.64%

			Cdk16 Ubfd1 Kti12 Cacybp Map2k2 Dap Pak1ip1 Mrpl35 Trim27 Cks1b Fgfbp1 Dnajc25 Pnpt1 Arpc2 Gusb Gatad2a U2af2 Hnrnpf Vdac1 Adat3 Nars2 Coro1c Ap2a1 Pgk1 Tmsb10 Capzb Rsl24d1 Ercc3 Kcnk6 Gale Snd1 Ran Lsm5 Psmb7 Nploc4 Samm50 Arpc4 Rer1 Sidt1 Gnai2 Pla2g5		
yellow	546	T cell activation, adaptive immune response, leukocyte differentiation, lymphocyte differentiation, B cell activation, mononuclear cell proliferation, leukocyte cell-cell adhesion, interferon-gamma production, positive regulation of cytokine biosynthetic process, gamma-delta T cell activation, interleukin-4 production, toll-like receptor signaling pathway, mast cell activation	Ppfia4 Atp8b2 Grap Ncf1 Gpnmb Celf2 Tespa1 Cybb Kcnab2 Fyb Lmo2 Pcdh15 Tnfsf8 P2ry13 Gpr171 Rasal3 Sit1 Dok3 Ceacam16 Zfp382 Ifi203 Pik3ap1 Ikzf1 Nup62-il4i1 Rorc Fermt3 Clec4n Magi2	76	13.92%
